# Impact of Partial Gelatinization on Structure, Physicochemical and Enzymatic Digestion Properties of Rice Starch Used for Rice Noodle-Making

**DOI:** 10.3390/polym17223003

**Published:** 2025-11-12

**Authors:** Bin Teng, Chen Zhang, Hui Wang

**Affiliations:** 1 Institute of Rice Research, Anhui Academy of Agricultural Sciences, Hefei 230031, China; ricebreeder@163.com; 2 College of Life Science, Anhui Agricultural University, Hefei 230036, China

**Keywords:** rice noodles, partially gelatinized starch, digestive characteristics, structural properties, physicochemical properties

## Abstract

Currently, information remains limited regarding how controlled gelatinization alters structure and functional properties of rice starch intended for rice noodle-making. The objective of this research is to understand the effect of partial gelatinization on structural and physicochemical attributes of rice starch from an elite variety Zhenguiai specifically for rice noodle production and their relations to digestion properties. Starch samples partially gelatinized at 60–76 °C were analyzed for crystalline fraction, amylose content (AC), swelling power, gelatinization properties and *in vitro* enzymatic digestibility, and compared with native starch. The results demonstrated substantial variations in relative crystallinity (RC), AC, swelling power, gelatinization transition temperatures and gelatinization enthalpy (Δ*H*). As the partial gelatinization temperature increased, the proportion of rapidly digestible starch (RDS) rose, whereas the contents of slowly digestible starch (SDS) and resistant starch (RS) declined. Correlation analyses between starch digestibility and other properties indicated that RDS was significantly positively correlated with both the onset (r = 0.936, *p* < 0.05) and peak (r = 0.895, *p* < 0.05) gelatinization temperatures but negatively correlated with RC (r = −0.954, *p* < 0.01), AC (r = −0.888, *p* < 0.05), and Δ*H* (r = −0.992, *p* < 0.01). Furthermore, RS demonstrated positive correlations with AC (r = 0.872, *p* < 0.05) and Δ*H* (r = 0.974, *p* < 0.01) while showing negative correlation with gelatinization onset temperature (r = −0.971, *p* < 0.01). Additionally, SDS exhibited a significant positive correlation with RC (r = 0.838, *p* < 0.05). These findings identify potential applications of partial gelatinization in guiding the development of modified starches with optimized physicochemical and digestibility properties, such as maintaining high levels of AC and RS, for the production of premium rice noodles.

## 1. Introduction

Rice (*Oryza sativa* L.) is a fundamental staple cereal crop that sustains the dietary needs of billions of people worldwide. As a convenient and nutritious food product, rice noodles are widely consumed across South Asia, including South China, Thailand, Malaysia, Indonesia and the Philippines. Typically, rice noodles are produced using high-amylose (>25%) *indica* rice varieties [1,2], as a higher amylose content facilitates the formation of a stable gel network structure [3]. Based on processing techniques and product characteristics, rice noodles can be broadly categorized into two groups: flat and extruded rice noodles [4]. Owing to their gluten-free nature and amylose levels, rice noodles are considered to offer considerable health benefits, particularly for individuals with celiac disease or gluten sensitivity [5,6,7].

The quality of rice noodles is primarily determined by the physicochemical properties of rice starch, which play a crucial role in the formation of the noodles’ structural network [8,9]. However, due to the lack of gluten proteins, native rice starch exhibits poor gel-forming ability and is therefore unsuitable for direct use in rice noodle production [5]. Additionally, native starch has limited application because of its instability under processing conditions, such as changes in temperature, shear force, and pH. To address these limitations, physical modification techniques are commonly employed to enhance the functionality of rice flour or starch for noodle preparation. Among these, hydrothermal treatments—such as annealing and heat-moisture treatment—are widely applied to improve starch quality [10,11,12,13]. These Hydrothermal treatments help stabilize starch granules, potentially enhancing the formation of a cohesive network during processing [14].

Hydrothermal treatment of rice starch may modify its granular and molecular structure, and subsequently lead to changes in multi-functional properties. Numerous prior studies have focused on examining the effect of annealing and heat-moisture treatments on the physicochemical properties and *in vitro* digestibility of rice starch [14,15,16,17,18,19]. The heat-moisture treatment and annealing have been proved to increase the gelatinization temperature [20,21], limit swelling, and enhance starch paste stability [22]. While existing studies have explored gelatinization effects, the specific influence of partial gelatinization on the structural and functional attributes of rice starch used in noodle manufacturing requires further comprehensive investigation.

Partial gelatinization of starch commonly occurs in the production of extruded rice noodles. In conventional processes, partially gelatinizing the rice starch is an essential modification step before extruding dough into noodles. Gelatinization is a key physical transformation process that critically influences the functional properties of starch. When heated in water, starch granules absorb water and swell. As water penetrates their amorphous regions, the crystalline structure is disrupted, causing the loss of birefringence—a clear indicator of gelatinization [23,24]. Starch granules exposed to varying thermal conditions undergo differential gelatinization, thereby influencing their functionality [25,26,27,28]. Previous reports revealed that heating-induced changes to starch structure can modulate postprandial metabolism [18,25,29]. Gelatinized starch may become more susceptible to enzymatic hydrolysis due to the breakdown of its granular structure [24]. Based on *in vitro* amylase digestion kinetics, starch can be classified into three fractions: rapidly digested (RDS), slowly digested (SDS), and resistant starch (RS) [30]. Among these, SDS is particularly important for preventing and managing non-insulin-dependent diabetes and obesity [19,31,32].

Several studies have investigated the properties of partially gelatinized starches derived from glutinous rice and corn [25,26,27,28]. However, there is currently no available data regarding the impact of partial gelatinization on the properties of starch specifically used for rice noodles. Thus, the present study aimed to elucidate the effect of controlled gelatinization on crystalline structure (X-ray diffraction pattern and relative crystallinity), amylose content, swelling power, as well as the gelatinization and *in vitro* digestibility properties of rice starch isolated from a high-quality rice cultivar (Zhenguiai) widely used in rice noodle-making. In addition, the relationship between starch nutritional fractions (RDS, SDS, and RS) and structural and physicochemical characteristics of rice starch was examined. The findings of this study may provide valuable insights for optimizing gelatinization conditions to improve the digestibility and physicochemical properties of rice starch in rice noodle manufacturing.

## 2. Materials and Methods

### 2.1. Plant Material

This study employed Zhenguiai rice, a traditional variety cultivated extensively in Southeast China, particularly Guangxi province, and valued for rice noodle production. Field experiments were conducted during the standard 2023 growing season (May to September) at the experimental farm of the Anhui Academy of Agricultural Sciences in Hefei, China. Crop management adhered to standard agronomic practices for paddy rice, including routine disease and insect control protocols. Following harvest, mature grains were air-dried, stored at ambient temperature for three months, and subsequently used for starch granule isolation.

### 2.2. Isolation of Native Starch

The native starch extraction followed an alkaline steeping protocol adapted from Teng et al. [33]. Milled brown rice was subjected to 48 h immersion in 0.2% (*w*/*v*) sodium hydroxide solution at ambient temperature. The softened grains were manually disrupted using mortar and pestle homogenization. Sequential wet sieving was performed through 100, 200, and 400 μm mesh screens to eliminate fibrous components. Subsequent purification involved (1) alkaline washing (0.2% NaOH) with 3000× *g* centrifugation for 10 min, (2) three cycles of deionized water resuspension followed by identical centrifugation parameters, and (3) careful removal of proteinaceous surface layers after each centrifugation. Final processing included two ethanol dehydration steps, ambient air-drying, and particle size standardization through 100 μm mesh screening.

### 2.3. Preparation of Partially Gelatinized Starches

Partially gelatinized starch was prepared following an adapted protocol from Fu et al. [27]. Native rice starch (2.0 g aliquots) was homogenized with 20 mL deionized water to prepare aqueous dispersions at ambient conditions. These suspensions underwent controlled thermal treatment in a shaking water bath (300 rpm) at incremental temperatures (60, 64, 68, 72, and 76 °C; the highest temperature serving as complete gelatinization reference) for 15 min. After thermal processing, all samples were immediately cooled to room temperature and subjected to 5000× *g* centrifugation for 10 min. Subsequent purification involved two ethanol washing cycles followed by convective drying at 40 °C for 36 h. The resulting granular starch products (designated PG-60 through PG-76 based on processing temperature) were mechanically pulverized and fractionated through a 100 μm mesh sieve prior to characterization.

### 2.4. X-Ray Diffraction Analysis

Crystalline structure analysis of rice starch samples was conducted on a Bruker D8 Advance X-ray diffractometer (Bruker AXS, Karlsruhe, Germany) operated at 40 kV and 200 mA. To ensure hydration equilibrium, all specimens were preconditioned for 7 days in a desiccator containing saturated NaCl solution, which generates a stable 75% relative humidity environment. Diffraction patterns were acquired across the 2θ range of 3–40° with a resolution of 0.02° and a dwell time of 0.8 s per step. Quantitative crystallinity determination was implemented through the two-phase deconvolution approach [34] using Microcal Origin 10.0 software (Northampton, MA, USA).

### 2.5. Differential Scanning Calorimetry Analysis

The gelatinization behavior of rice starch variants was investigated through differential scanning calorimetry (Netzsch 200-F3, Selb, Germany) under controlled hydration conditions. Precisely 3.0 mg of anhydrous starch was homogenized with ultrapure water at a 1:3 (*w*/*w*) ratio (9 μL) in hermetically sealed DSC crucibles. After 4 h equilibration at 20 °C to ensure uniform water distribution, thermal profiling was executed from 20 to 120 °C with a linear heating gradient of 10 °C/min, using an empty crucible as reference. All measurements were conducted in triplicate to ensure statistical reliability.

### 2.6. Measurement of Amylose Content and Swelling Power

Amylose quantification was conducted through iodine complexation spectrophotometry following the protocol of Konik-Rose et al. [35]. with optimization. Briefly, 10 mg starch aliquots were subjected to lipid removal using 5 mL 85% (*v*/*v*) methanol at 65 °C for 1 h with periodic agitation, followed by centrifugation (3000× *g*, 5 min). The delipidized starch was subsequently solubilized in 5 mL UDMSO solvent system (90% DMSO/10% 6 M urea, *v*/*v*) at 95 °C for 1 h with intermittent mixing. For chromogenic reaction, 1 mL of the solubilized starch was reacted with 1 mL iodine reagent (0.2% I_2_/2% KI, *w*/*v*) and diluted to 50 mL, with absorbance measured at 620 nm (UV-1800, Shimadzu, Kyoto, Japan). Amylose content (AC) was derived from a standard curve generated with Sigma-Aldrich amylose/amylopectin mixtures. Swelling behavior was evaluated according to Cai et al. [36] with procedural adjustments. Precisely 60 mg anhydrous starch was hydrated in 3 mL ultrapure water, heated at 95 °C for 30 min with gentle mixing, then equilibrated to room temperature for 30 min. After centrifugation (8000× *g*, 15 min), the swollen pellet mass was determined gravimetrically. All analyses included triplicate measurements to ensure methodological reproducibility. Swelling power was calculated as

Swelling power (g/g) = (sediment weight)/(amount of dry starch − amount of dried supernatant).(1)

### 2.7. In Vitro Digestion Analysis of Starch

The enzymatic digestibility of native starch was evaluated through an *in vitro* simulated digestion protocol adapted from Carciofi et al. [37]. Specifically, 20 mg starch samples were hydrolyzed in 4 mL reaction medium containing: 20 mM sodium phosphate buffer (pH 6.0), 6.7 mM NaCl, 0.01% NaN_3_, 2.5 mM CaCl_2_, 4 U porcine pancreatic α-amylase (PPA, Sigma-Aldrich A3176, St. Louis, MO, USA), and 4 U Aspergillus niger amyloglucosidase (AAG, Megazyme E-AMGDF, Bray Business Park Southern Cross Road Bray, Co., Wicklow, Ireland). Incubation proceeded at 37 °C with orbital shaking (1000 rpm) for kinetic time points (20 min and 120 min). Reactions were quenched by ice-cold acidification (480 μL 0.1 M HCl) and ethanol precipitation (2 mL 50%), followed by centrifugation (14,000× *g*, 5 min) to collect hydrolysates. Released glucose was quantified using GOPOD enzymatic assay (Megazyme K-GLUC, Bray Business Park Southern Cross Road Bray, Co., Wicklow, Ireland) at 510 nm. Based on hydrolysis kinetics, starch fractions were classified as follows: Rapidly digestible starch (RDS): hydrolyzed within 20 min; Slowly digestible starch (SDS): hydrolyzed during 20–120 min; Resistant starch (RS): remaining undigested after 120 min. All assays included triplicate measurements with coefficient of variation <5%.

### 2.8. Statistical Analysis

Statistical evaluations were implemented with IBM SPSS Statistics (Version 20.0, Armonk, NY, USA). Multiple comparisons among group means were assessed through Duncan’s least significant difference test at α = 0.05 significance threshold. Bivariate correlation analysis elucidated potential associations between starch digestibility parameters and gelatinization-related physicochemical characteristics.

## 3. Results and Discussion

### 3.1. X-Ray Diffraction Spectrum of Starch

**Figure 1:**
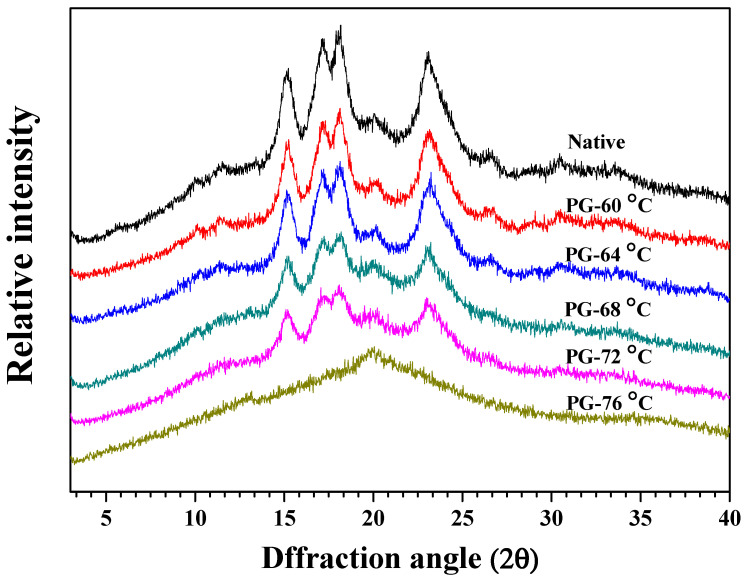
XRD spectra of native and partially gelatinized starches.

The crystalline architecture of Zhenguiai rice starch was systematically characterized through X-ray diffraction analysis (Figure 1), revealing temperature-dependent polymorphic transitions. Native starch exhibited prototypical A-type crystallinity, manifested by prominent reflections at 15° and 23° 2θ accompanied by characteristic shoulder peaks at 17–18° 2θ, consistent with cereal starch signatures [33]. Partial gelatinization (60–72 °C) preserved the A-type diffraction pattern albeit with progressive peak broadening, suggesting crystalline domain fragmentation without polymorphic conversion. Complete gelatinization at 76 °C resulted in complete peak disappearance, confirming transition to an amorphous state. This structural evolution aligns with the two-stage gelatinization model of Waigh et al. [38], wherein thermal energy first induces helical dissociation in crystalline lamellae, followed by cooperative unwinding of double helices—a process concurrently involving water penetration into amorphous regions, granular deformation, blocklet dispersion, and amylose leaching.

The hierarchical organization of starch crystallites arises from the systematic arrangement of double helices formed by amylopectin’s flexible ‘A’ chains [24]. Our thermal treatment experiments demonstrated progressive disassembly of this crystalline architecture, with relative crystallinity (RC) decreasing from 29.36% to 0.05% across gelatinization temperatures (Table 1). Generally, it is suggested that differences in RC between starches are influenced by many factors, including crystal size, amount of crystalline regions which is influenced by amylopectin content and amylopectin chain length, orientation of the double helices within the crystalline domains, and the extent of interaction between double helices [33,36,39]. The present results indicated that double helices of the starches gelatinized at 68–76 °C were less compactly packed and or less well arranged.

### 3.2. Amylose Content and Swelling Power

Amylose content plays an important role in starch internal quality. Apparent amylose contents of the partially gelatinized rice starches are presented in Table 1. The AC of native starch was 25.37% meeting the general requirement of rice noodle production [2]. Before the heating temperature increased to 68 °C, no significant differences in AC were observed between the partial gelatinized and native starch samples (Table 1). When the heating temperature was above 68 °C, ACs of the treated starches decreased from 24.18% to 21.18% (Table 1). The observed amylose release phenomena primarily originate from the thermal-induced structural disintegration of starch granules, encompassing both granule swelling and crystalline domain disruption during thermal processing [33,36].

Upon thermal treatment in aqueous environments, starch undergoes crystalline phase transition, where liberated hydroxyl groups on amylose and amylopectin chains form extensive hydration shells, causing an increase in granule swelling [24]. The swelling initiation of starch granules predominantly occurs within the bulk amorphous phase characterized by higher molecular mobility, followed by subsequent expansion into the constrained amorphous domains adjacent to crystalline lamellae. In the present study, the increase in partial gelatinization temperature might result in decreased swelling powers of Zhenguiai rice starch. As seen in Table 1, starches heated at 60 °C exhibited a remarkably lower mean value (10.50) of SP than that of the native starch samples (16.28), and a similar trend has also been observed for corn starches [27]. When the gelatinization temperature increased from 64 °C to 68 °C, no significant differences (*p* < 0.05) in SP between the PG samples were observed. The SP values reached a plateau value at 72–76 °C with a SP range of 8.91–9.10 (Table 1). The degree of starch granule expansion correlates with the structural disorganization of polymer chains within the granule matrix, particularly in regions exhibiting reduced crystallinity [24,33]. The SP has been suggested to be mainly influenced by amylose content, amylopectin molecular structure and crystalline structure [33,36]. Previous studies showed that SP is positively correlated with RC but negatively correlated with AC [33,36]. In this paper, the decreased SPs of starches were clearly associated with the reduced RCs but not ACs (Table 1), strongly indicating that the changes in SP should be attributed to the variations in RC. Sasaki and Matsuki [40] demonstrated that starches exhibiting greater swelling capacity are typically characterized by an elevated proportion of long-chain amylopectin branches, with structural polymorphs in amylopectin directly correlating with differential swelling behaviors, which underscores the necessity for future investigations into how partial gelatinization modulates the architectural configuration of amylopectin molecules.

### 3.3. Gelatinization Properties

The gelatinization characteristics of rice starches were quantitatively assessed using differential scanning calorimetry, with key thermodynamic parameters including onset (*T*_o_), peak (*T*_p_), and conclusion (*T*_c_) temperatures, transition temperature range (Δ*T*, *T*_c_–*T*_o_), and enthalpy change (Δ*H*) systematically presented in Table 2. The *T*_o_, *T*_p_ and *T*_c_ of native rice starch were 61.50 °C, 68.83 °C, and 75.57 °C, respectively, in agreement with the range of gelatinization temperature for various rice starches [2,33,36,41]. The present results showed that there existed significant differences in *T*_o_, *T*_p_, *T*_c_, Δ*T* and Δ*H* between the partially gelatinized and control samples. The gelatinization transition temperatures increased as the heating temperature (60–72 °C) increased, while an opposite trend was observed for Δ*T* and Δ*H* (Table 2). The changes that take place during gelatinization have been attributed to water availability, crystal stability within granules, difference between amorphous and crystalline regions, and glass transition related progressive melting [29]. The gelatinization process is fundamentally driven by four interdependent mechanisms including water accessibility to starch granules, crystalline domain destabilization, phase transition dynamics between amorphous and crystalline regions, and glass transition-mediated sequential melting [29]. As suggested by previous studies, gelatinization temperatures are negatively correlated with AC [33,36]. In the current study, the results showed that the starch samples treated with higher heat temperature exhibited higher gelatinization transition temperatures (Table 1 and Table 2), in agreement with previous results [33,36]. Upon further heating, starch polymers become more mobile, reduce or lose their interpolymer interactions, and starch granules disrupt (no thermal transitions at 76 °C), as shown in Table 2. The thermal behavior during gelatinization is intrinsically modulated by crystalline domain organization, particularly through the spatial arrangement of amylopectin short chains (degree of polymerization 6–12) within starch granules [42]. Structural analysis revealed that starch variants with lower gelatinization onset exhibit reduced crystalline perfection and diminished crystallite integrity compared to their high-temperature counterparts, a phenomenon directly linked to subtle amylopectin structural variations [43]. Therefore, post-gelatinization residual crystallites, characterized by heterogeneous microcrystalline domains with differential mechanical stability, may account for the observed variations in *T*_o_, *T*_p_ and *T*_c_ temperatures during thermal transitions.

DSC enthalpies of native and partially gelatinized starches showed little changes until 64 °C, and then gradually dropped to a very low level at 72 °C (Table 2). In addition, no enthalpies were observed at 76 °C (Table 2), indicating the absence of crystalline domains. As suggested by Tester and Morrison [43], Δ*H* serves as a quantitative indicator of amylopectin crystalline organization, encompassing both crystallite density and spatial distribution. Consequently, the observed enthalpy variations in this study likely stem from treatment-induced modifications in crystalline architecture, particularly through differential partial gelatinization protocols (Table 1 and Table 2). In other hand some researchers suggested that Δ*H* measures double-helix breakdown in both crystalline and amorphous regions, not just crystallinity loss [16,44]. Under hydrating conditions, gelatinization initiates as a swelling-mediated process. The absorption of water into both bulk amorphous regions and inter-crystalline zones induces localized expansion, mechanically compromising the integrity of amylopectin crystallites. This weakening effect causes the arrangement of starch molecular chains to become loose, making the originally tight helical structure (e.g., the double helix of amylopectin) require more thermal energy to completely dissociate. As a result, the structural disruption ultimately enhances the thermal energy required for helix dissociation [24]. Thus, the experimental data suggest that double-helix formation is likely compromised between 64 and 76 °C, attributable to weakened interactions between amylose and amylopectin chains (Table 1 and Table 2).

### 3.4. In Vitro Starch Digestibility

**Figure 2:**
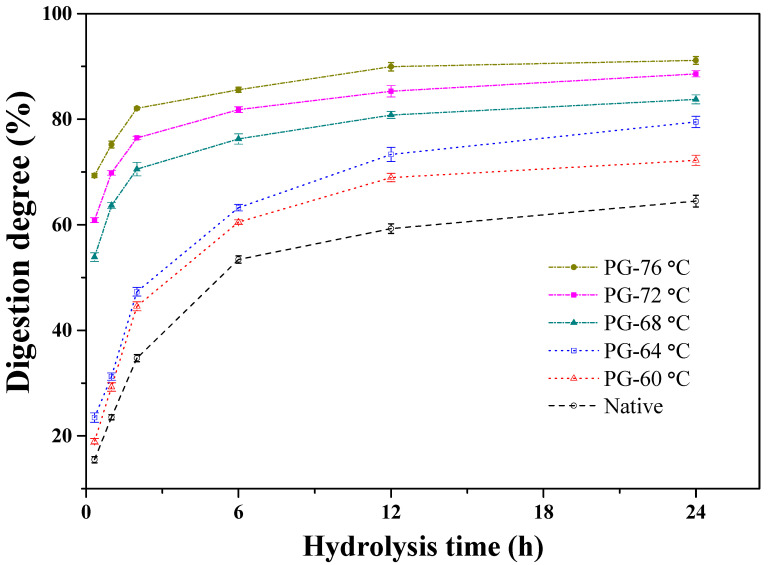
Enzymatic digestion properties of native and partially gelatinized starches. Vertical bars represent mean values ± standard deviations (n = 3). The digestion degrees are expressed as the percentage of glucose released from native starches by PPA and AAG. PPA, porcine pancreatic-amylase; AAG, Aspergillus niger amyloglucosidase.

The *in vitro* digestive behaviors of native and partially gelatinized starch samples (PG-60, PG-64, PG-68, PG-72, PG-76) were comparatively assessed using PPA and AAG to simulate small intestinal starch hydrolysis. Digestibility kinetics under varying thermal treatments are quantitatively illustrated in Figure 2. The enzymatic hydrolysis exhibited a temperature-dependent enhancement, with higher gelatinization degrees correlating with accelerated degradation kinetics. The hydrolysis process followed a distinct biphasic pattern: an initial rapid phase (0–6 h) was succeeded by a gradual deceleration, reaching plateau conditions by 24 h. Distinct hydrolysis kinetics were observed among starch variants, with the PG-76 starch demonstrating rapid saturation (~2 h) compared to native starch (12 h to plateau). Thermal treatments ≥68 °C significantly enhanced enzymatic susceptibility, yielding accelerated degradation profiles. All samples achieved complete digestion after 24 h incubation. Thermal treatment induces progressive disruption of hydrogen bonding networks within starch granules, culminating in granular swelling and structural disintegration [24]. This gelatinization-mediated structural modification significantly enhances enzyme accessibility to starch polymers, as evidenced by the accelerated digestion kinetics observed (Figure 2). Considering that the partially gelatinized samples contained a wide range of crystallinity (0.05–27.29%) (Table 1), the residual crystallinity of starch does not necessarily reflect the amount of enzyme-resistant starch residues. For the PG-76 starch sample, most of swollen starch granules were completely disrupted by excess heat and mechanical agitation, resulting in transformation to a continuous amorphous structure, whereas other partially gelatinized samples might contain swollen starch granules that were not completely disrupted. Therefore, the granular integrity, regardless of swelling level, might also play an important role in determining the maximum level of enzymatic digestion.

**Figure 3:**
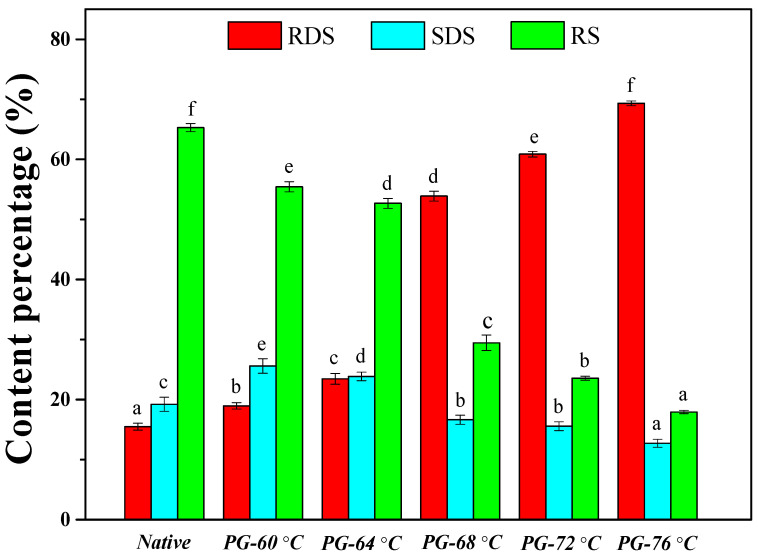
Starch nutritional fractions of native and partially gelatinized rice starches. Data (Mean ± standard deviation, n = 3) carrying different letters on the bars with same color indicate a significant difference at the 0.05 probability level. RDS, rapidly digestible starch; SDS, slowly digestible starch; RS, resistant starch.

Figure 3 delineates the dynamic partitioning of starch nutritional fractions (RDS/SDS/RS) across native and partially gelatinized variants, wherein thermal treatments induced statistically significant compositional modulation (*p* < 0.05). Under excess-water gelatinization conditions, temperature elevation exhibited a dose-dependent enhancement of RDS yield, peaking at 69.33% upon complete gelatinization (76 °C) accompanied by reciprocal declines in SDS and RS fractions (Figure 3). In this research, the thermal window of 60–76 °C triggers starch gelatinization, a multi-stage process characterized by ordered granule disorganization. Chung et al. [25] reported that partially gelatinized starch exhibited digestibility intermediates between native and fully gelatinized forms, with enzymatic hydrolysis efficiency demonstrating sigmoidal dependence on gelatinization completion (melting enthalpy integration). Of this study, in excess-water conditions, starch granules exhibited biphasic water absorption: preferential hydration of the background region persists below 60 °C, followed by a structural transition where excess water migrates into amorphous lamellae. This phase shift, occurring near 60 °C, coincides with a marked reduction in crystallinity, likely triggered by double helix dissociation. Such behavior aligns with Cameron and Donald’s [45] model of swelling mode transformation from radial to tangential expansion. In this study, thermal treatment of rice starch at 76 °C yielded maximal RDS content (Figure 3), corroborating Miao et al.’s [46] observation that cooking induces SDS/RS reduction. This phenomenon stems from granule structural reorganization: heat disrupts both inter- and intra-chain hydrogen bonds, facilitating granule swelling and subsequent disassembly. Progressive gelatinization thereby enhances enzymatic accessibility through chain disentanglement. Zhang et al. [47] demonstrated that thermal processing induces complete A-type starch granule amorphization, thereby abolishing its inherent slow-digestion characteristic. Complementary findings by Benmoussa et al. [48] revealed that amylopectin branch architecture governs the enzymatic susceptibility of thermally modified starch. In this study, our experimental data revealed a negative correlation between amylose content and RDS formation (Figure 3, Table 1), demonstrating that amylose depletion enhances α-amylase digestibility post-gelatinization, which is consistent with Bornet et al. [49], who observed an inverse relationship that appears between amylose content and the degree of glycemic response to processed starchy food.

### 3.5. Starch Nutritional Fractions in Relation to Structural and Physicochemical Properties of Starch

Table 3 presents Pearson correlation analyses linking starch nutritional fractions (RDS, SDS, RS) with structural/physicochemical properties across the native and partially gelatinized starch samples. As shown in Table 3, the relative crystallinity was significantly negatively correlated with RDS (r = −0.954, *p <* 0.01), but significantly positively correlated with SDS (r = 0.838, *p <* 0.05) and RS (r = 0.944, *p <* 0.01). Similarly to relative crystallinity, AC showed negative correlation with RDS (r = −0.888, *p <* 0.05), but positive correlations with SDS (r = 0.809) and RS (r = 0.872, *p <* 0.05). The SP and *T*_c_ were both found to have no significant correlations with the RDS, SDS, and RS, respectively. Gelatinization onset temperature (*T*_o_) represents melting of the weakest crystallites, and is more sensitive to crystallite perfection on annealing [24]. The present study indicated that *T*_o_ was significantly and positively correlated with RDS (r = 0.936, *p <* 0.05) and significantly and negatively correlated with RS (r = −0.971, *p <* 0.01) but not significantly correlated with SDS (r = −0.563). However, starch nutritional fractions had no significant correlations with *T*_p_ and *T*_c_, with the exception of the RDS which had significant correlation with *T*_p_ (r = 0.895, *p <* 0.05). Rapidly digestible starch was significantly negatively correlated with Δ*T* (r = −0.957, *p* < 0.01) and Δ*H* (r = −0.992, *p* < 0.01). Contrary to RDS, the opposite trend was observed for RS, which exhibited significant positive correlations with Δ*T* (r = 0.989, *p* < 0.01) and Δ*H* (r = 0.974, *p* < 0.01). In addition, the SDS had no significant correlations with Δ*T* and Δ*H*. Collectively, these findings elucidate the mechanistic linkage between structural-functional alterations in partially gelatinized starches and their consequent modulation of enzymatic digestibility of rice starch used for rice noodle-making.

## 4. Conclusions

In this study, the partial gelatinization treatment exerted a significant influence on the structural and physicochemical properties of rice noodle starches. The findings in the present study showed that partial gelatinization induced comprehensive alterations in starch properties, encompassing attenuated X-ray diffraction signals, diminished crystallinity ratios, modified amylose distributions, reduced swelling capacities, shifted gelatinization temperature profiles, and altered enthalpy transition behaviors. These differences might stem from differential molecular chain interactions within the amorphous and crystalline regions. This study also showed that thermal processing induced divergent digestibility patterns between native and partially gelatinized rice starches, with escalating gelatinization temperatures (particularly beyond 68 °C) markedly elevating RDS proportions while diminishing RS levels. Correlation analysis revealed the relationships between starch nutritional fractions (RDS, SDS, and RS) and structural and physicochemical properties of native and partially gelatinized starches. This research demonstrates that controlled partial gelatinization offers a strategic approach to obtain tailored physicochemical and digestibility profiles of rice starch specifically used for rice noodle-making.

## Figures and Tables

**Table 1:** Relative crystallinities, amylose contents and swelling powers of native and partially gelatinized rice starches.

Zhenguiai	Relative Crystallinity (%)	Amylose Content (%)	Swelling Power
*Native*	29.36 ± 0.51 ^f^	25.37 ± 0.40 ^d^	16.28 ± 0.47 ^d^
*PG-60*	27.29 ± 0.54 ^e^	25.15 ± 0.28 ^d^	10.50 ± 0.33 ^c^
*PG-64*	23.99 ± 0.56 ^d^	24.92 ± 0.23 ^d^	9.99 ± 0.10 ^b^
*PG-68*	15.22 ± 0.77 ^c^	24.18 ± 0.43 ^c^	9.61 ± 0.14 ^b^
*PG-72*	12.94 ± 0.38 ^b^	23.42 ± 0.66 ^b^	9.10 ± 0.04 ^a^
*PG-76*	0.05 ± 0.01 ^a^	21.18 ± 0.35 ^a^	8.91 ± 0.25 ^a^

Data (Mean ± standard deviation, n = 3) in the same column with different letters indicate the significance at the 0.05 probability level.

**Table 2:** Gelatinization properties of native and partially gelatinized starches.

Zhenguiai	Thermal Parameter				
	*T*_o_ (°C)	*T*_p_ (°C)	*T*_c_ (°C)	Δ*T* (°C)	Δ*H* (J/g)
*Native*	61.50 ± 0.17 ^a^	68.83 ± 0.15 ^a^	75.57 ± 0.15 ^a^	13.07 ± 0.25 ^d^	12.42 ± 0.43 ^c^
*PG-60*	65.30 ± 0.10 ^b^	69.07 ± 0.23 ^b^	76.53 ± 0.25 ^b^	10.23 ± 0.15 ^c^	11.91 ± 0.61 ^c^
*PG-64*	66.13 ± 0.21 ^c^	69.00 ± 0.17 ^ab^	76.80 ± 0.36 ^b^	9.67 ± 0.21 ^b^	11.69 ± 0.34 ^c^
*PG-68*	69.37 ± 0.15 ^d^	70.77 ± 0.15 ^c^	76.57 ± 0.15 ^b^	6.20 ± 0.20 ^a^	5.42 ± 0.36 ^b^
*PG-72*	72.90 ± 0.20 ^e^	74.30 ± 0.26 ^d^	78.93 ± 0.42 ^c^	5.03 ± 0.42 ^a^	2.67 ± 0.10 ^a^
*PG-76*	ND	-	-	-	-

Data (Mean ± standard deviation, n = 3) in the same column with different letters indicate the significance at the 0.05 probability level. ND indicates no data. *T*_o_, onset temperature; *T*_p_, peak temperature; *T*_c_, conclusion temperature; Δ*T*, gelatinization range (*T*_c_–*T*_o_); Δ*H*, enthalpy of gelatinization.

**Table 3:** Pearson correlation coefficients for the relationship between starch nutritional fractions and structural and physicochemical properties in native and partially gelatinized rice starches.

Property	RDS	SDS	RS
RCD ^a^	−0.954 **	0.838 *	0.944 **
AC ^a^	−0.888 *	0.809	0.872 *
SP ^a^	−0.674	0.274	0.760
*T*_o_ ^b^	0.936 *	−0.563	−0.971 **
*T*_p_ ^b^	0.895 *	−0.738	−0.878
*T*_c_ ^b^	0.755	−0.426	−0.789
Δ*T* ^b^	−0.957 **	0.586	0.989 **
Δ*H* ^b^	−0.992 **	0.815	0.974 **

* and ** indicate that the correlation is significant at *p* < 0.05 and *p* < 0.01 level, respectively. RDS, rapidly digestible starch; SDS, slowly digestible starch; RS, resistant starch; AC, amylose content; RCD, relative crystal degree; SP, swelling power; *T*_o_, onset temperature; *T*_p_, peak temperature; *T*_c_, conclusion temperature; Δ*T*, gelatinization temperature; Δ*H*, gelatinization enthalpy. ^a^ and ^b^ indicate the n = 6 and n = 5 (no values detected when gelatinized at 76 °C), respectively.

## Data Availability

The original contributions of this study are detailed in the Supplementary Materials. Further inquiries may be directed to the corresponding author.

## Supplementary Materials

The following supporting information can be downloaded at: https://www.mdpi.com/article/10.3390/polym17223003/s1.
